# YWHAE silencing induces cell proliferation, invasion and migration through the up-regulation of CDC25B and MYC in gastric cancer cells: new insights about YWHAE role in the tumor development and metastasis process

**DOI:** 10.18632/oncotarget.13381

**Published:** 2016-11-16

**Authors:** Mariana Ferreira Leal, Helem Ferreira Ribeiro, Juan Antonio Rey, Giovanny Rebouças Pinto, Marília Cardoso Smith, Caroline Aquino Moreira-Nunes, Paulo Pimentel Assumpção, Leticia Martins Lamarão, Danielle Queiroz Calcagno, Raquel Carvalho Montenegro, Rommel Rodriguez Burbano

**Affiliations:** ^1^ Disciplina de Genética, Departamento de Morfologia e Genética, Universidade Federal de São Paulo, São Paulo, Brazil; ^2^ Departamento de Ortopedia e Traumatologia, Universidade Federal de São Paulo, São Paulo, Brazil; ^3^ Núcleo de Pesquisas em Oncologia, Hospital Universitário João de Barros Barreto, Belém, Brazil; ^4^ Instituto de Ciências Biológicas, Universidade Federal do Pará, Belém, Brazil; ^5^ Laboratorio de Oncogenética Molecular, Hospital Universitario La Paz, Madrid, Spain; ^6^ Departmento de Biomedicina, Universidade Federal do Piauí, Parnaíba, Brazil; ^7^ Departamento de Fisiologia e Farmacologia, Faculdade de Medicina, Universidade Federal do Ceará, Fortaleza, Brazil; ^8^ Laboratório de Testes de Ácidos Nucleicos, Fundação Centro de Hemoterapia e Hematologia do Pará, Belém, Brazil; ^9^ Laboratório de Biologia Molecular, Hospital Ophir Loyola, Belém, Brazil

**Keywords:** gastric cancer, cancer development, YWHAE, CDC25B, MYC

## Abstract

We previously observed reduced YWHAE (14-3-3ε) protein expression in a small set of gastric cancer samples. YWHAE may act as a negative regulator of the cyclin CDC25B, which is a transcriptional target of MYC oncogene. The understanding of YWHAE role and its targets is important for the better knowledge of gastric carcinogenesis. Thus, we aimed to evaluate the relationship among YWHAE, CDC25B, and MYC *in vitro* and *in vivo*. For this, we analyzed the YWHAE, CDC25B, and MYC expression in *YWHA*-silenced, *CDC25B*-silenced, and *MYC*-silenced gastric cancer cell lines, as well as in gastric cancer and non-neoplastic gastric samples. In gastric cancer cell lines, *YWHAE* was able to inhibit the cell proliferation, invasion and migration through the reduction of *MYC* and *CDC25B* expression. Conversely, *MYC* induced the cell proliferation, invasion and migration through the induction of *CDC25B* and the reduction of *YWHAE*. Most of the tumors presented reduced YWHAE and increased CDC25B expression, which seems to be important for tumor development. Increased MYC expression was a common finding in gastric cancer and has a role in poor prognosis. In the tumor initiation, the opposite role of *YWHAE* and *CDC25B* in gastric carcinogenesis seems to be independent of *MYC* expression. However, the inversely correlation between *YWHAE* and *MYC* expression seems to be important for gastric cancer cells invasion and migration. The interaction between YWHAE and MYC and the activation of the pathways related to this interaction play a role in the metastasis process.

## INTRODUCTION

Gastric cancer (GC) is one of the most common causes of cancer death in the World [1]. Advanced GC presents few treatment options and a poor prognosis, which is in part due to the tumor recurrence, invasion or metastasis. The relative five-year survival rate is below than 20% [2]. It is still necessary to determine the key molecular factors involved in GC initiation and progression.

In eukaryotes, the 14-3-3s are part of a highly conserved protein family. Seven *14-3-3* genes encode nine protein isoforms, including two phosphorylated forms (α and δ) [3, 4]. The 14-3-3 proteins are mainly dimeric within the cell and are able to bind several sites within a target or act as a bridge between proteins [5–7]. 14-3-3 proteins can interact with hundreds of proteins, including cdc25 phosphatase [4, 5, 7, 8]. The precise function of 14-3-3 proteins is not fully understand. However, these proteins seem to play a role as molecular scaffolds [4] and regulate different biologic processes, including apoptosis, mitogenic signal transduction, and cell cycle (for reviews, see references [5, 9, 10]).

Deregulated expression of 14-3-3 proteins has been detected in some GC proteomic studies [11–14]. We previously observed reduced YWHAE, also called 14-3-3ε, protein expression in a small set of GC specimens [15]. Reduced YWHAE expression has also been described in other cancers [16–18], suggesting that this protein may play a role as a tumor suppressor.

YWHAE acts as a negative regulator of CDC25 [19, 20]. CDC25 phosphatases play a key role in cell cycle proliferation. CDC25B seems to present oncogenetic properties [21] and its overexpression was described previously in GC [22–25]. The subcellular localization of CDC25B can be controlled by its association with 14-3-3 proteins. CDC25B subcellular location might contribute to stall the cell cycle at the G2 phase following DNA damage [26–29].

At the transcription level, CDC25B is also a target of MYC and they may mediate MYC-induced cell cycle activation and/or apoptosis [30]. A correlation between CDC25B and MYC immunoreactivity was earlier described in GC [25]. *MYC*, located at 8q24, is a key oncogene in gastric carcinogenesis [31]. We previously demonstrated that MYC mRNA and protein increased expression is a common finding in GC samples [32–35] and some preneoplastic gastric lesions [36, 37] from a Brazilian population. Our research group also showed MYC expression increases during gastric carcinogenesis in a nonhuman primate model [38]. Moreover, we described several genetic and epigenetic alterations involving *MYC* gene in GC samples or GC cell lines, including chromosome 8 trisomy [32, 39–43], gene or 8q24 amplification [32–36, 39, 44–46], gene insertion [47], promoter hypomethylation [34] and point mutations [34]. However, the understanding of MYC targets is important for the better knowledge of its role in gastric carcinogenesis and may help in the development of new anticancer therapies.

Based on our previous findings, we hypothesized that MYC or CDC25B up-regulation may induce YWHAE down-regulation in GC or YWHAE down-regulation would induce CDC25B up-regulation in this neoplasia, which would also contribute to MYC overexpression. In this study, we aimed to better understand the relationship of the expression of these genes *in vivo* and *in vitro*. For this, we simultaneously evaluated the YWHAE, CDC25B, MYC and mRNA and protein expression in GC cell lines and in a large set of GC and paired non-neoplastic gastric samples. Additionally, we investigated the possible associations between gene/protein expression and clinical variables.

## RESULTS

### mRNA and protein expression in gastric cell lines

**Figure 1:**
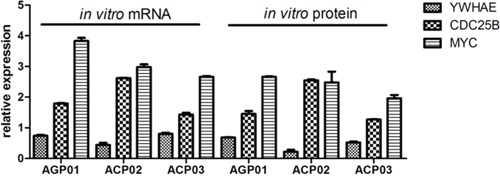
*YHWAE*, *CDC25B* and *MYC* mRNA and protein expression in gastric cancer cell lines in relation to non-neoplastic cells MNP01 non-neoplastic cells were used as a calibrator. Values of median and IQR are shown.

We firstly accessed the mRNA and protein expression of *YWHAE*, *CDC25B* and *MYC* in GC cell lines in relation to the non-neoplastic MNP01 cells (Figure 1). GC cell lines presented a reduced *YWHAE* mRNA and protein expression in relation to MNP01 cells [mRNA median (interquartile range, IQR): 0.71 (0.31); protein median (IQR): 0.52 (0.40); respectively]. On the other hand, the GC cell lines presented an increased *CDC25B* [mRNA median (IQR): 1.79 (1.15); protein median (IQR): 1.45 (1.24); respectively] and *MYC* [mRNA median (IQR): 2.98 (1.13); protein median (IQR): 2.48 (0.66); respectively] expression.

### YWHAE silencing induces GC cell proliferation, invasion and migration

**Figure 2:**
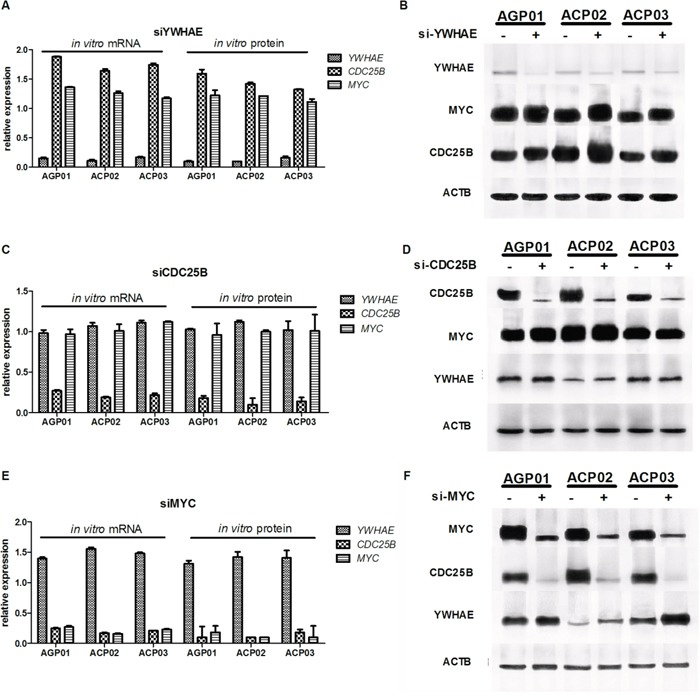
Effect of gene silencing in gene and protein expression in gastric cancer cell lines **A.** *YWHAE* silencing induced *MYC* and *CDC25B* increased expression in GC cell lines. **B.** GC cells with (+) or without (-) *YWHAE* silencing, equal amounts of whole cell extracts were analyzed by western blot with the indicated antibodies. **C.** *CDCD25B* silencing did not alter *MYC* and *YHWAE* expression in GC cell lines. **D.** GC cells with (+) or without (-) *CDC25B* silencing; Equal amounts of whole cell extracts were analyzed by western blot with the indicated antibodies. **E.** *MYC* silencing induced the reduction of *CDC25B* expression and increasing of *YHWAE* expression in GC cell lines. **F.** GC cells with (+) or without (-) *MYC* silencing; equal amounts of whole cell extracts were analyzed by western blot with the indicated antibodies. siRNA control-transfected cells were used as a calibrator. Values of median and IQR are shown.

**Figure 3:**
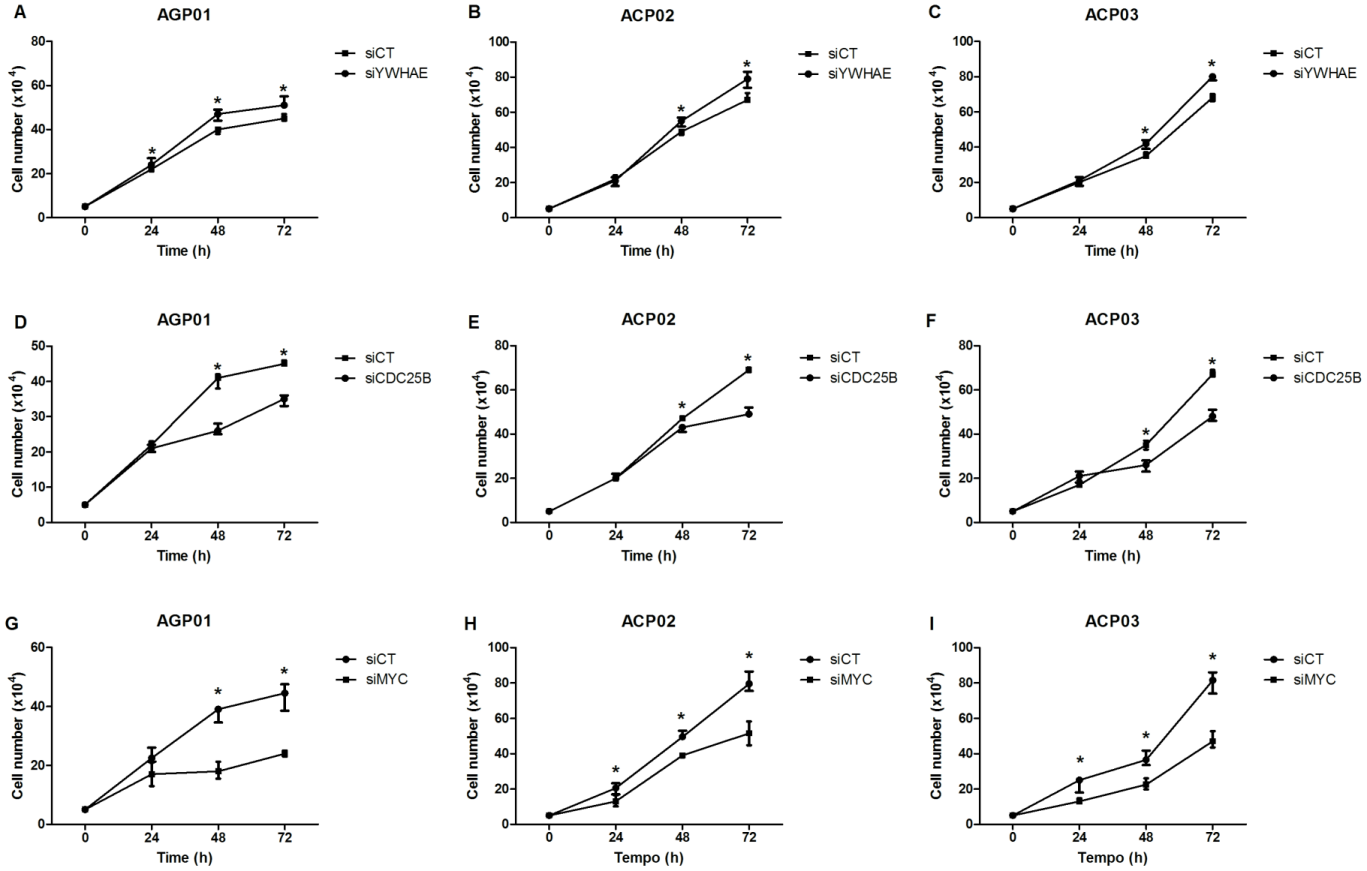
Effect of gene silencing in gastric cancer cell proliferation **A.** Effect of *YWHAE* silencing in AGP01 cell line. **B.** Effect of *YWHAE* silencing in ACP02 cell line. **C.** Effect of *YWHAE* silencing in ACP03 cell line. **D.** Effect of *CDC25B* silencing in AGP01 cell line. **E.** Effect of *CDC25B* silencing in ACP02 cell line. **F.** Effect of *CDC25B* silencing in ACP03 cell line. **G.** Effect of *MYC* silencing in AGP01 cell line. **H.** Effect of *MYC* silencing in ACP02 cell line. **I.** Effect of *MYC* silencing in ACP03 cell line. Cell counting was measured after 24, 48, and 72 h of silencing. ^*^p<0.05, significant difference between controls and silenced cells by Mann-Whitney test. Values of median and IQR are shown. siCT: controls cells; siYWHAE: cells with *YWHAE* silencing; siCDC25B: cells with *CDC25B* silencing; siMYC: cells with *MYC* silencing.

**Figure 4:**
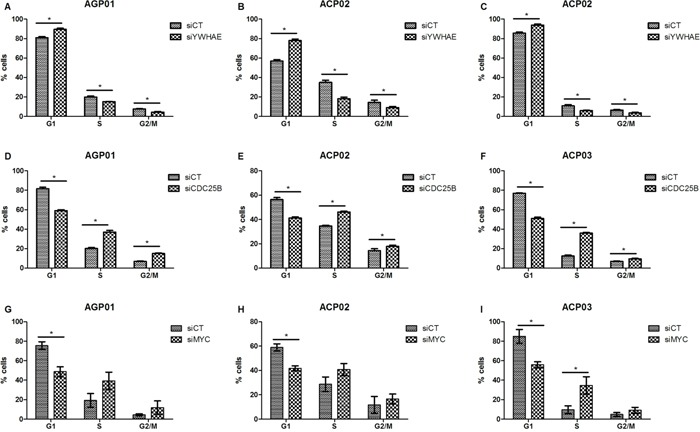
Effect of si-RNA silencing on cell cycle progression of gastric cancer cell lines **A.** Effect of *YWHAE* silencing in AGP01 cell line. **B.** Effect of *YWHAE* silencing in ACP02 cell line. **C.** Effect of *YWHAE* silencing in ACP03 cell line. **D.** Effect of *CDC25B* silencing in AGP01 cell line. **E.** Effect of *CDC25B* silencing in ACP02 cell line. **F.** Effect of *CDC25B* silencing in ACP03 cell line. **G.** Effect of *MYC* silencing in AGP01 cell line. **H.** Effect of *MYC* silencing in ACP02 cell line. **I.** Effect of *MYC* silencing in ACP03 cell line. The cell cycle analysis was performed at 72 h after transfection. ^*^p<0.05, significant difference between controls and silenced cells by Mann-Whitney test. Values of median and IQR are shown. siCT: controls cells; siYWHAE: cells with *YWHAE* silencing; siCDC25B: cells with *CDC25B* silencing; siMYC: cells with *MYC* silencing.

siRNA decresead *YWHAE* expression in more thand 80% in ACP03 and in more than 90% in AGP01 and ACP02 cell lines (Figure 2A–2B). Furthermore, *YWHAE* silencing induced cell proliferation (*p*<0.05, for all comparisions; Figure 3A–3C) and induces cell arrest by increase of G1/G0 cells and decrease in cells in S and G2/M (*p*<0.05, for all comparisions; Figure 4A–4C).

**Figure 5:**
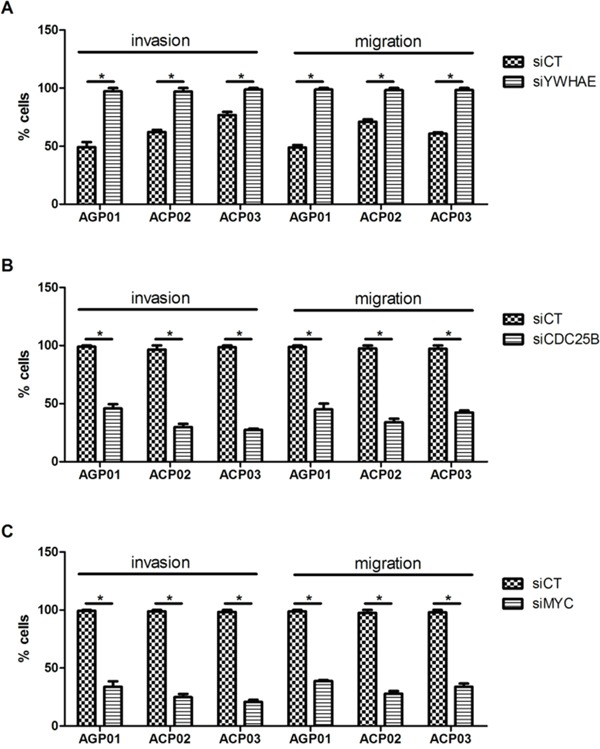
Effect of si-RNA silencing on gastric cancer cell lines invasion and migration **A.** Effect of *YWHAE* silencing in GC cell line. **B.** Effect of *CDC25B* silencing in GC cell line. **C.** Effect of *MYC* silencing in GC cell line. ^*^p<0.05, significant difference between controls and silenced cells by Mann-Whitney test. Values of median and IQR are shown. siCT: controls cells; siYWHAE: cells with *YWHAE* silencing; siCDC25B: cells with *CDC25B* silencing; siMYC: cells with *MYC* silencing.

*YWHAE* silencing induced cell invasion and migration in all gastric cancer cell lines (*p*<0.05, for all comparisions; Figure 5A).

### YWHAE regulates CDC25B and MYC in GC cells

**Figure 6:**
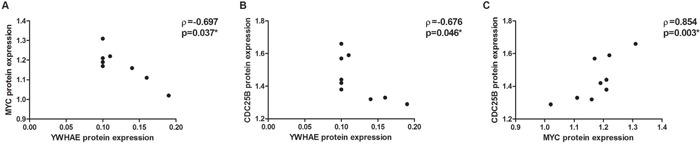
Correlation between protein expression in *YWHAE*-silenced AGP01, ACP02 and ACP03 gastric cancer cell lines **A.** YWHAE and MYC. **B.** YWHAE and CDC25B. **C.** MYC and CDC25B. Correlation coefficients and p-values of Spearman correlation test are shown. ^*^p<0.05, significant correlation by Spearman correlation test.

*YWHAE* silencing induced *CDC25B* up-regulation by at least 1.6-fold in all GC cell lines (Figure 2A) and a slight *MYC* up-regulation by approximately 1.25-fold in all GC cell lines (Figure 2A). At protein level, YWHAE expression was inversely correlated with MYC (ρ=-0.697; *p*=0.037; Figure 6A) and CDC25B expression (ρ=-0.676; *p*=0.046; Figure 6B). MYC and CDC25B protein expression was also correlated in *YWHAE* silencing cells (ρ=0.854; *p*=0.003; Figure 6C).

Then, we silenced *CDC25B* and *MYC* in GC cell to evaluate the effect of these genes in GC cells, as well as the effect on *YWHAE* expression. siRNA caused a reduction in *CDC25B* expression of more than 80% in all three GC cell lines (Figure 2C–2D). Although *CDC25B* silencing induced a significant decrease in cell proliferation (Figure 3D–3F), control cells were accumulated in G1 in relation to si-*CDC25B* cells (*p*<0.05, for all comparisions; Figure 4D–4F). *CDC25B* silencing inhibits GC cells invasion and migration (Figure 5B). However, *CDC25B* silencing did not induced alteration in *MYC* or *YWHAE* mRNA and protein expression (Figure 2B).

Conversely, siRNA caused a reduction in *MYC* expression of more than 70% in all three studied GC cell lines (Figure 2E–2F). The cell lines also presented significant alterations concerning cell proliferation. All cell lines presented a significant decrease in proliferation after 48 h and 72 h of silencing *MYC* (*p*<0.05, for all comparisons; Figure 3G–3I). Moreover, ACP02 and ACP03 also showed a reduction in cell proliferation after 24 h of *MYC* silencing (*p*<0.05, for all comparisons; Figure 3G–3I). After 72 h, *MYC* silencing leads to alterations in the GC cell cycle. Slight variations were observed among GC cell lines (Figure 4G–4I). Control cells were accumulated in G1, and there was a statistical significance when comparing control cells to siRNA-*MYC* cells (*p*<0.05, for all comparisions), as detected in si-*CDC25B* cells. Furthermore, *MYC* silencing inhibed GC cells invasion and migration (*p*<0.05, for all comparisions; Figure 5C).

**Figure 7:**
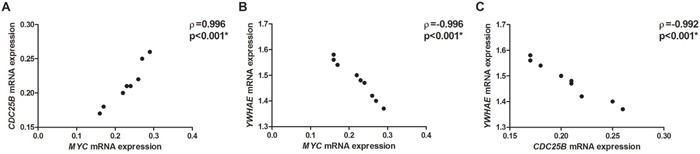
Correlation between the studied genes mRNA expression in *MYC*-silenced AGP01, ACP02 and ACP03 gastric cancer cell lines **A.** *MYC* and *CDC25B*. **B.** *MYC* and *YWHAE*. **C.** *CDC25B* and *YWHAE*. Correlation coefficients and p-values of Spearman correlation test are shown. ^*^p<0.05, significant correlation by Spearman correlation test.

We next evaluate whether *MYC* may regulate *YWHAE* and *CDC25B* in gastric cells. *MYC* silencing induced *CDC25B* down-regulation by at least 4-fold in all GC cell lines (Figure 2E). Conversely, *MYC* silencing up-regulated *YWHAE* by approximately 1.5-fold in all GC cell lines (Figure 2E). Considering all cell lines, we observed that *MYC* expression was directly correlated with *CDC25B* expression (ρ=0.996; *p*<0.001; Figure 7A) and inversely correlated with *YWHAE* expression (ρ=-0.996; *p*<0.001; Figure 7B). Moreover, a strong inverse correlation was detected between *YWHAE* and *CDC25B* expression (ρ=-0.992; *p*<0.001; Figure 7C). MYC and YWHAE protein expression was also inversely correlated (ρ=-0.703; *p*<0.033).

### YWHAE, CDC25B, and MYC expression in gastric samples

**Figure 8:**
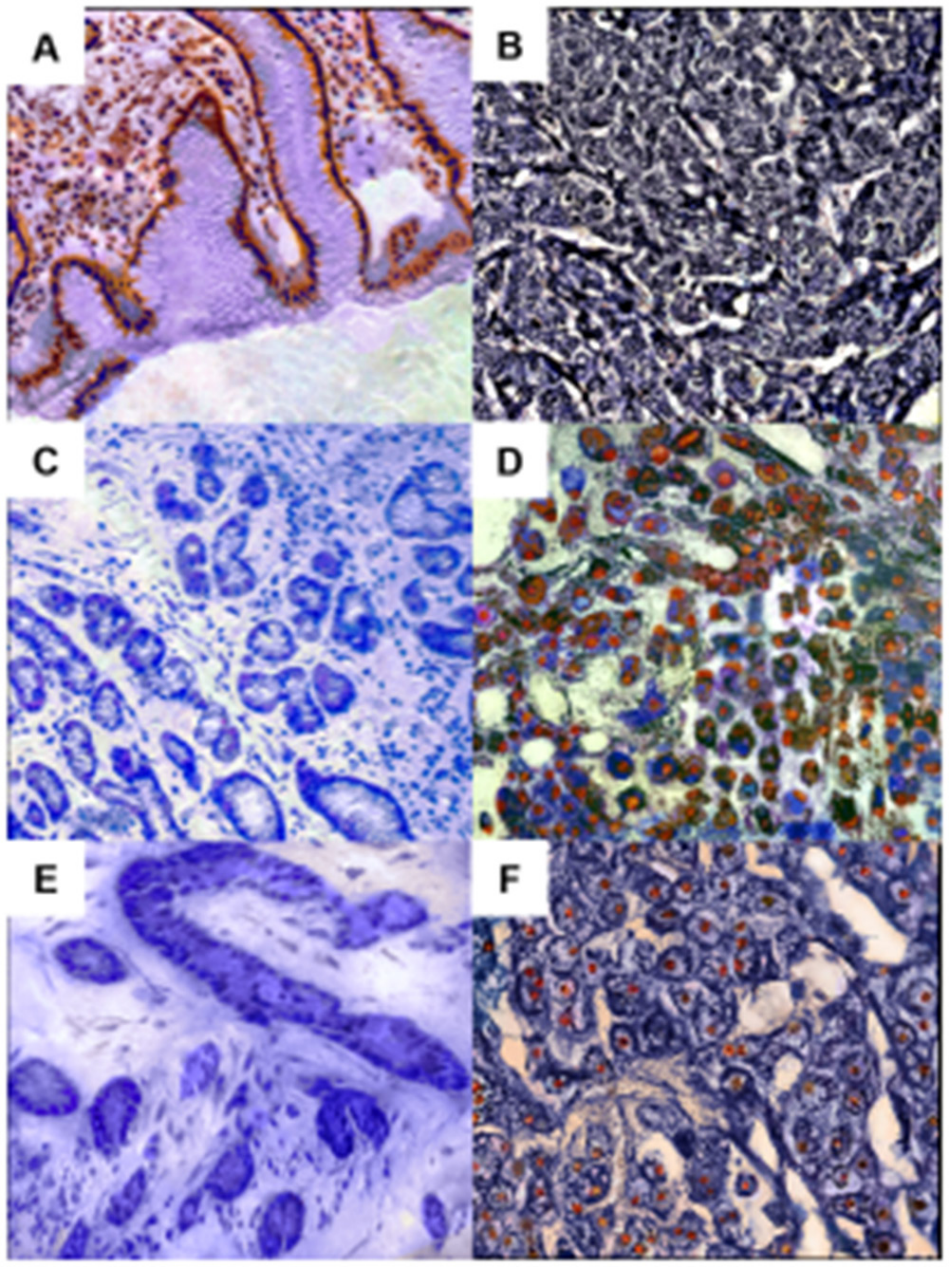
YWHAE, CDC25B and MYC immunoreactivity in gastric tissue samples **A.** Non-neoplastic gastric mucosa showing cytoplasmic YWHAE staining. **B.** intestinal-type gastric cancer cells without YWHAE immunoreactivity. **C.** Non-neoplastic gastric tissue without CDC25B immunoreactivity. **D.** Diffuse-type gastric cancer presenting nuclear and cytoplasmic CDC25B immunoreactivity. **E.** Gastric mucosa without MYC immunoreactivity. **F.** Intestinal-type gastric cancer presenting nuclear immunoreactivity of MYC.

YWHAE immunoreactivity was detected in the cytoplasm of lymphocytes and non-neoplastic gastric cells (Figure 8A). YWHAE immunoreactivity was observed in only 6 (4.7%) of the gastric tumor tissue samples (Figure 8B). CDC25B was detected in the nuclei and cytoplasm of 128 (99.2%) GC samples (Figure 8D). Nuclear immunoreactivity for MYC was detected in 117 (90.7%) GC samples (Figure 8F). Conversely, nonatypical gastric cells did not present CDC25B and MYC immunoreactivity (Figure 8C and 8E).

**Figure 9:**
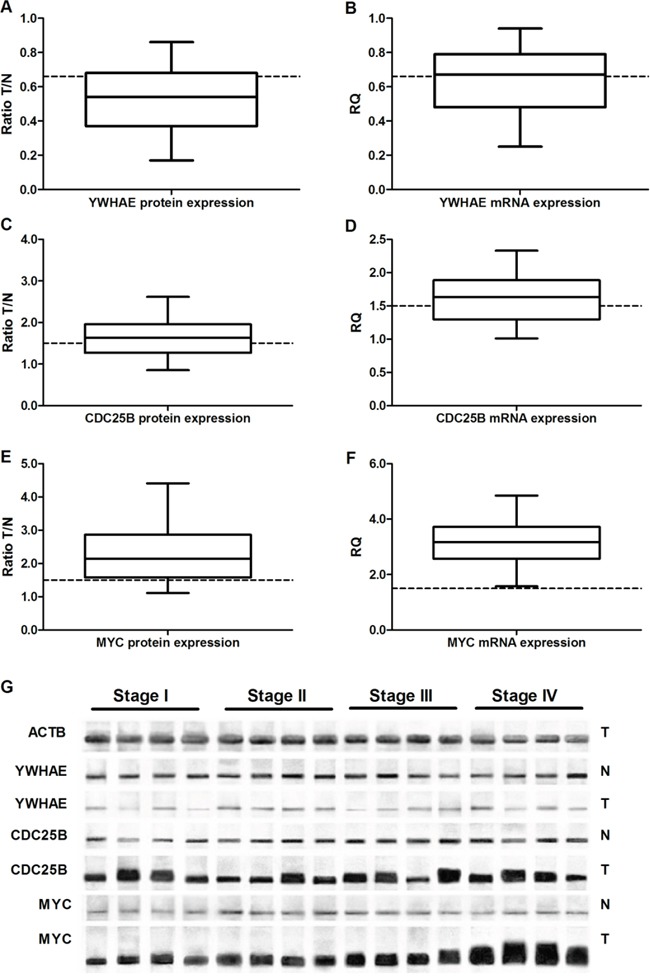
Protein and gene expression in gastric cancer. **A.** YWHAE protein expression **B.** *YHWAE* mRNA expression. **C.** CDC25B protein expression. **D.** *CDC25B* mRNA expression. **E.** MYC protein expression. **F.** *MYC* mRNA expression. **G.** Representative image of Western-blot. In all graphs, the expression in gastric tumors was normalized by matched non-neoplastic gastric tissue. T: tumor sample; N: normal mucosa sample; RQ: relative quantification. The whiskers indicate the minimum and maximum values. The dotted lines representes the 1.5 fold-change.

Down-regulation (at least 50% decrease in expression) of YWHAE protein and mRNA was detected in 89 (69%) and 61 (47.3%) GC samples, respectively (Figure 9A–9B, G).

Conversely, protein and mRNA levels of CDC25B were increased more than 1.5-fold (increment of at least 50% in expression) in 72 (55.8%) and 77 (59.7%) GC samples, respectively (Figure 9C–9D, G). In addition, MYC protein and mRNA expression increased more than 1.5-fold in 101 (78.3%) and 129 (100%) GC samples, respectively, in comparison to paired non-neoplastic gastric specimens (Figure 9E–9F, G).

**Figure 10:**
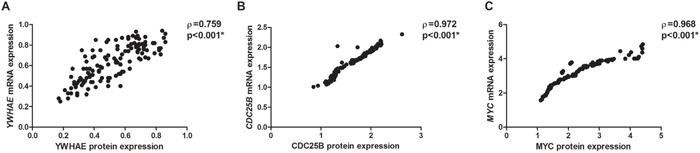
Correlation between protein and mRNA expression in gastric cancer samples **A.** YWHAE. **B.** CDC25B. **C.** MYC. Correlation coefficients and p-values of Spearman correlation test are shown. ^*^p<0.05, significant correlation by Spearman correlation test.

MYC (*p*=0.002 and 0.001, respectively) and YWHAE (*p*=0.007 and <0.001, respectively) immunoreactivity was associated with higher protein and mRNA levels in GC samples. A strong correlation was observed between mRNA and protein expression for YWHAE (ρ=0.759; *p*<0.001; Figure 10A), CDC25B (ρ=0.972; *p*<0.001; Figure 10B), and MYC (ρ=0.968; *p*<0.001; Figure 10C).

**Figure 11:**
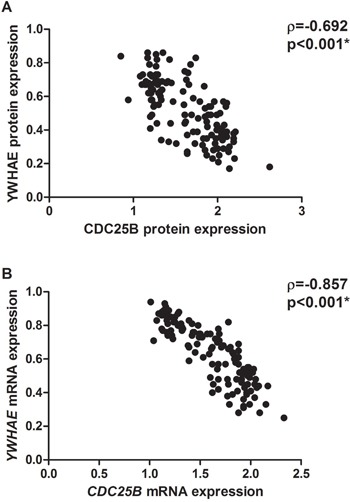
Correlation between YWHAE and CDC25B in all the studied gastric cancer samples **A.** protein expression. **B.** mRNA expression. Correlation coefficients and p-values of Spearman correlation test are shown. ^*^p<0.05, significant correlation by Spearman correlation test.

An inverse correlation was detected between YWHAE and CDC25B protein (ρ=-0.692; *p*<0.001; Figure 11A) and mRNA (ρ=-0.857; *p*<0.001; Figure 11B) expression. Moreover, 122 (94.6%) of tumors presented CDC25B immunoreactivity and lack of YWHAE immunostaining.

MYC protein and mRNA expression was not correlated with CDC25B or YWHAE expression (*p*>0.05 for all analyses). However, 117 (90.7%) of the tumors presented both MYC and CDC25B immunoreactivity, and 111 (86%) of the tumors presented MYC immunoreactivity and lack of YWHAE immunostaining.

**Figure 12:**
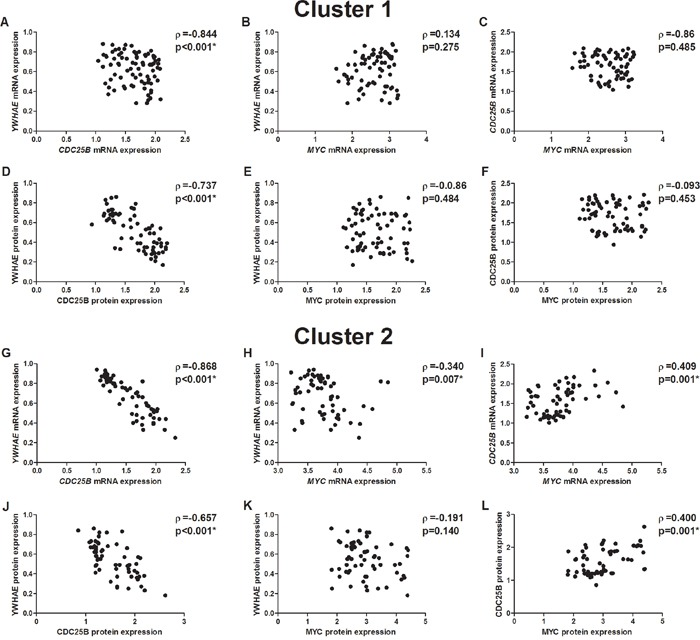
Correlation between mRNA and protein expression in two cluster of gastric cancer samples **A.** *YWHAE* and *CDC25B* mRNA expression in the cluster 1. **B.** *YWHAE* and *MYC* mRNA expression in the cluster 1. **C.** *CDC25B* and *MYC* mRNA expression in the cluster 1. **D.** YWHAE and CDC25B protein expression in the cluster 1. **E.** YWHAE and MYC protein expression in the cluster 1. **F.** CDC25B and MYC protein expression in the cluster 1. **G.** *YWHAE* and *CDC25B* mRNA expression in the cluster 2. **H.** *YWHAE* and *MYC* mRNA expression in the cluster 2. **I.** *CDC25B* and *MYC* mRNA expression in the cluster 2. **J.** YWHAE and CDC25B protein expression in the cluster 2. **K.** YWHAE and MYC protein expression in the cluster 2. **L.** CDC25B and MYC protein expression in the cluster 2. Correlation coefficients and p-values of Spearman correlation test are shown. ^*^p<0.05, significant correlation by S Spearman correlation test.

We used the K-means clustering method to group samples based on their gene expression similarities. In the K-means clustering method, we observed the presence of two clusters. In both clusters, a strong inverse correlation was also detected between *YWHAE* and *CDC25B* mRNA (ρ=-0.844, *p*<0.001, Figure 12A for “Cluster 1” and ρ=-0.868, *p*<0.001, Figure 12G for “Cluster 2”) and protein (ρ=-0.737, *p*<0.001, Figure 12D for “Cluster 1” and ρ=-0.657, *p*<0.001, Figure 12J for “Cluster 2”) expression. In one of the clusters (named “Cluster 2”), *MYC* mRNA and protein expression was directly correlated with *CDC25B* expression (ρ=0.409, *p*=0.001, Figure 12I; ρ=0.400, *p*=0.001, Figure 12L; respectively) and inversely correlated with *YWHAE* mRNA expression (ρ=-0.340, *p*=0.007, Figure 12H). Interestingly, “Cluster 2” was composed of samples with the highest *MYC* expression, in which the mRNA level was increased by at least 3.2-fold and protein level was increased by at least 1.8-fold.

### Association between YWHAE, CDC25B, and MYC expression with clinicopathological features in GC

Clinicopathological variables and YWHAE, CDC25B, and MYC expression are shown in Table 1. YWHAE protein expression was reduced in tumors of male in relation to tumors of females (*p*=0.046; Table 1).

**Table 1:** Clinicopathological variables and gene expression in GC

	YWHAE immunoreactivity	YWHAE protein	*YWHAE* mRNA	CDC25B immunoreactivity	CDC25B protein	*CDC25B* mRNA	MYC immunoreactivity	MYC protein	*MYC* mRNA
Variable	N	N (%) of positive cases	*p* value^a^	Ratio T/N [median (IQR)]	*p* value^b^	RQ [median(IQR)]	*p* value^b^	N (%) of positive cases	*P* value^a^	Ratio T/N [median(IQR)]	*p* value^b^	RQ [median(IQR)]	*P* value^b^	N (%) of positive cases	*p* value^a^	Ratio T/N [median (IQR)]	*p* value^b^	RQ [median (IQR)]	*p* value^b^
**Gender**																			
Female	45	2 (4.4)	0.652	0.57 (0.26)	0.046^*^	0.64 (0.32)	0.592	45 (100)	0.651	1.71 (0.73)	0.349	1.68 (0.61)	0.376	41 (91.1)	0.589	2.11 (1.55)	0.687	3.22 (1.38)	0.953
Male	84	4 (4.8)		0.49 (0.33)		0.69 (0.30)		83 (98.8)		1.61 (0.66)		1.61 (0.60)		76 (90.5)		2.16 (1.18)		3.11 (1.09)	
**Onset**																			
< 45 years	34	3 (8.8)	0.187	0.41 (0.27)	0.006^*^	0.49 (0.21)	<0.001^*^	34 (100)	0.736	1.96 (0.50)	0.003^*^	1.88 (0.40)	0.005^*^	2 (94.1)	0.339	1.80 (0.92)	0.013^*^	3.04 (1.27)	0.048^*^
≥ 45 years	95	3 (3.2)		0.57 (0.29)		0.71 (0.23)		94 (99.2)		1.45 (0.61)		1.57 (0.52)		85 (89.5)		2.23 (1.27)		3.28 (1.01)	
**Tumor location**																		
Cardia	50	1 (2)	0.247	0.57 (0.25)	0.379	0.68 (0.22)	0.489	50 (100)	0.612	1.64 (0.64)	0.625	1.61 (0.53)	0.383	43 (86)	0.126	1.87 (1.27)	0.088	3.06 (1.32)	0.197
Non-cardia	79	5 (6.3)		0.49 (0.34)		0.66 (0.35)		78 (98.7)		1.62 (0.72)		1.68 (0.64)		74 (93.7)		2.20 (1.16)		3.23 (0.93)	
**Histological type**																		
Diffuse	62	3 (4.8)	0.622	0.39 (0.25)	<0.001^*^	0.58 (0.33)	0.019^*^	62 (100)	0.519	1.84 (0.72)	0.041^*^	1.78 (0.62)	0.027^*^	52 (83.9)	0.010^*^	2.20 (1.13)	0.786	3.21 (0.92)	0.854
Intestinal	67	3 (4.5)		0.63 (0.22)		0.72 (0.26)		66 (98.5)		1.43 (0.57)		1.52 (0.50)		65 (97)		2.11 (1.40)		3.09 (1.22)	
**Stage**																			
Early	12	0 (0)	0.550	0.36 (0.28)	0.027^*^	0.48 (0.19)	0.003^*^	12 (100)	0.907	1.83 (0.45)	0.102	1.80 (0.33)	0.046^*^	10 (83.3)	0.309	1.37 (0.60)	<0.001^*^	2.25 (1.25)	0.003^*^
Advanced	117	6 (5.1)		0.55		0.68		116		1.61		1.62		107		2.19		3.23					(0.29)		(0.29)		(99.1)		(0.68)		(0.60)		(91.5)		(1.24)		(1.03)	
**Tumor invasion**																		
T1/T2	42	0 (0)	0.089	0.42 (0.28)	0.006^*^	0.57 (0.23)	0.001^*^	42 (100)	0.674	1.79 (0.42)	0.004^*^	1.76 (0.33)	0.004^*^	36 (85.7)	0.151	1.63 (0.77)	<0.001^*^	2.59 (1.12)	<0.001^*^
T3/T4	87	6 (6.9)		0.58 (0.28)		0.72 (0.29)		86 (98.9)		1.35 (0.67)		1.48 (0.62)		81 (93.1)		2.38 (1.20)		3.40 (0.95)	
**Lymph node metastasis**																	
Absent	16	1 (6.3)	0.556	0.52 (0.27)	0.379	0.62 (0.27)	0.438	15 (93.8)	0.124	1.93 (0.65)	0.092	1.86 (0.58)	0.123	11 (68.8)	0.007^*^	1.42 (0.25)	<0.001^*^	2.36 (0.48)	<0.001^*^
Present	113	5 (4.4)		0.55 (0.31)		0.67 (0.32)		113 (100)		1.61 (0.65)		1.62 (0.59)		106 (93.8)		2.23 (1.21)		3.29 (0.97)	
**Distant metastasis**																		
Absent	70	3 (4.3)	0.576	0.57 (0.30)	0.134	0.67 (0.27)	0.970	69 (98.6)	0.543	1.63 (0.69)	0.620	1.63 (0.59)	0.712	58 (82.9)	<0.001^*^	1.67 (0.61)	<0.001^*^	2.63 (0.76)	<0.001^*^
Present	59	3 (5.1)		0.49 (0.32)		0.66 (0.36)		59 (100)		1.63 (0.68)		1.67 (0.64)		59 (100)		2.93 (0.96)		3.74 (0.44)	
*H. pylori*																			
Negative	13	0 (0)	0.522	0.39 (0.31)	0.109	0.57 (0.35)	0.315	13 (100)	0.899	1.76 (0.82)	0.134	1.72 (0.68)	0.127	12 (92.3)	0.654	2.17 (1.41)	0.879	3.02 (1.27)	0.656
Positive	116	6 (5.2)		0.55 (0.31)		0.67 (0.30)		115 (99.1)		1.62 (0.68)		1.63 (0.60)		105(90.5)		2.14 (1.30)		3.21 (1.16)	
**CagA**																			
Negative	46	0 (0)	0.066	0.54 (0.32)	0.611	0.66 (0.30)	0.508	45 (97.8)	0.357	1.63 (0.71)	0.526	1.63 (0.55)	0.665	41 (89.1)	0.435	2.08 (1.15)	0.789	3.17 (1.05)	0.904
Positive	83	6 (7.2)		0.56 (0.31)		0.70 (0.33)		83 (100)		1.64 (0.69)		1.65 (0.66)		76 (91.6)		2.18 (1.33)		3.17 (1.14)	
**EBV**																			
Negative	108	4 (3.7)	0.252	0.54 (0.30)	0.742	0.67 (0.30)	0.723	107 (99.1)	0.837	1.64 (0.69)	0.730	1.64 (0.57)	0.990	96 (88.9)	0.107	2.04 (1.14)	0.024^*^	3.08 (1.10)	0.025^*^
Positive	21	2 (9.5)		0.58 (0.36)		0.64 (0.34)		21 (100)		1.39 (0.68)		1.60 (0.66)		21 (100)		2.57 (1.71)		3.69 (1.19)	

a p value by ÷2 test;

b p value by Mann-Whitney test.

* p<0.05, significantly difference between groups.

** A tendency for different expression between groups.

N: number of samples; T/N: ratio of protein expression between neoplastic and matched non-neoplastic samples; RQ: relative quantification, in which the matched non-neoplastic sample was designated as a calibrator from each neoplastic samples; IQR: interquartile range; EBV: Epstein-Barr virus.

The cancer samples of patients with late-onset tumors presented higher YWHAE and MYC expression and lower CDC25B expression in relation to to early-onset GC samples (*p*<0.05 for all analyses; Table 1).

YWHAE protein and mRNA expression was reduced in diffuse-type GC (*p*<0.001 and 0.019, respectively; Table 1). Conversely, CDC25B protein and mRNA expression was increased in diffuse-type GC (*p*=0.041 and 0.027, respectively; Table 1). MYC immunoreactivity was more frequent in intestinal-type GC (*p*=0.010; Table 1).

Decreased YWHAE protein and mRNA expression was also associated with early-stage and T1/T2 tumors (*p*<0.05 for all comparisons; Table 1). In contrast, CDC25B protein and mRNA expression was higher in T1/T2 tumors in relation to T3/T4 tumors (*p*<0.05 for all analyses; Table 1). CDC25B protein expression was also increased in early-stage tumors in comparison to advanced-stage tumors (*p*=0.046; Table 1).

Increased MYC expression was detected in tumors with advanced stage, deeper invasion, lymph node and distant metastases and with EBV infection (*p*<0.05 for all analyses; Table 1). Moreover, MYC immunoreactivity was more frequent in tumors of patients with lymph node or distant metastases (*p*<0.05 for all analyses; Table 1).

**Figure 13:**
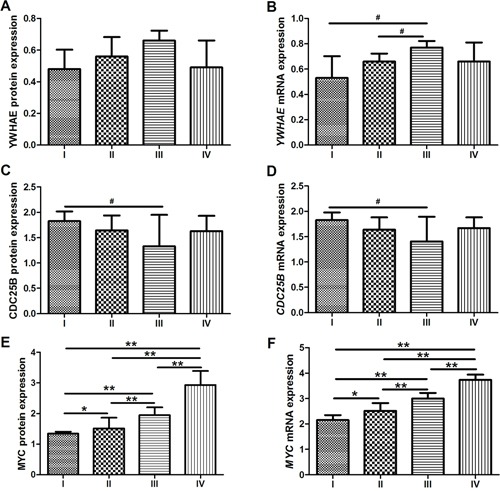
Protein and mRNA expression by tumor stage. **A.** YWHAE protein expression **B.** *YWHAE* mRNA expression. **C.** CDC25B protein expression. **D.** *CDC25B* mRNA expression. **E.** MYC protein expression. **F.** *MYC* mRNA expression. ^*^p<0.008, significant difference between groups (Mann-Whitney test followed by Bonferroni corrections); ^**^p<0.001, significant difference between groups (Mann-Whitney test followed by Bonferroni corrections); #*p*<0.05, difference between groups but not statistically significant after Bonferroni adjustment. Values of median and IQR are shown.

YWHAE expression seems to increase and CDC25B expression seems to reduce between stages I and III; however, we did not detect a significant change among tumor stages after Bonferroni adjustment (*p*>0.008; Figure 13A–13D). A gradual increase of MYC expression was detected in GC samples through tumor stages I to IV (*p*<0.008 for all analyses; Mann-Whitney test followed by Bonferroni correction; Figure 13E–13F).

The inverse correlation between YWHAE and CDC25 mRNA and protein expression was independent of the tumor stage, tumor invasion or ocurrence of lymph node and distant metastases (p<0.05; for all comparisons). Interestingly, *YWHAE* and *MYC* expression was directly correlated (ρ=0.361; *p*=0.002) and *CDC25B* and *MYC* expression was inversely correlated (ρ=-0.271; *p*=0.023) in tumors of patients without distant metastasis. However, in tumors of patients with distant metastasis or in stage IV GC, *MYC* and *CDC25B* mRNA expression was positively correlated (ρ=0.269; *p*<0.039) and MYC and CDC25B protein expression also tended to be correlated (ρ=0.248; *p*=0.058).

Moreover, the “Cluster 2” described above was associated with advanced GC stage, deeper tumor invasion (T stage), the occurrence of lymph node metastasis and the occurrence of distant metastases (*p*<0.05 for all analyses; Supplementary Table S1).

## DISCUSSION

Reduced *YWHAE* expression was described in different types of cancer, including lung [16], larynx [17], and brain [18] cancers. Our group also detected reduced expression of YWHAE in a smaller GC samples cohort [15]. Although these findings provide evidences to support classification of YWHAE as a tumor suppressor gene, the precise role of this gene in gastric carcinogenes was still unknown.

*In vitro*, we demonstrated that *YWHAE* acts as a tumor suppressor in GC inducing cell arrest and inhibiting cell invasion and migration through the down-regulation of *MYC* and *CDC25B*. On the other hand, *MYC* oncogene is able to induces GC cell proliferation, invasion and migration through the up-regulation of *CDC25B* and down-regulation of *YWHAE*. Our findings suggest that YWHAE and MYC may have a opposite role in GC cells. A previous study showed that the knockdown of another member of 14-3-3 family, the YWHAS, leads to increased expression of MYC protein [48]. Thus, other 14-3-3 family member can regulates *MYC* during gastric carcinogenesis.

In tissue samples, YWHAE immunoreactivity was detected in only approximately 5% of tumors and its 1.5-fold reduced protein and mRNA expression was observed in aproximately 70% and 50% of the studied tumors, respectively. Moreover, none of the tumors presented the YWHAE expression higher than the matched non-neoplastic gastric sample. This findings confirm that YWHAE may act as a tumor suppressor as observed *in vitro* and as described previously by our group in a smaller GC samples cohort [15]. On the contrary, CDCD25B immunoreactivity was observed in almost all tumors and its mRNA and protein expression was increased by at least 1.5-fold in more than 50% of the cases in relation to non-neoplastic samples. However, it is important to highlight that only two samples presented the ratio of CDC25B protein expression between tumor and corresponding non-neoplastic specimens below 1 in the studied cohort. This finding is in agreement with the oncogenic role of CDC25B in GC samples from our admixed population (mainly composed by European, Africans, and Amerindians [49]) as already described in GC samples from patients from East Asian [22–25].

Although direct silencing of *CDC25B* did not altered *YWHAE* expression, *CDC25B* and *YWHAE* expression were inversely correlated in *MYC* or *YWHAE* silenced cells, as well as in GC samples. YWHAE negatively regulates CDC25 [19, 20]. The hypothesis of CDC25B negative regulation by YWHAE in gastric carcinogenesis is also supported by our findings concerning the association of these genes or proteins expression and clinicopathological features. Reduced YWHAE and increased CDC25B expression was associated with early-onset GC, diffuse-type, T1/T2 stage, and early-stage tumors.

Our group previously described the association or tendency to association of reduced YWHAE expression with early-onset GC, diffuse-type, T1/T2 stage, and early-stage GC [15]. Thus, our findings reinforce that decreasing of YWHAE expression may be important for tumor initiation, especially in diffuse-type tumors an of early-onset. Early-onset GC presents distinct molecular and clinicopathological pattern in relation to late-onset tumors, which suggest that they are a two subsets of GC [50–52]. Furthermore, our results also supports that the intestinal and diffuse histological GC types follow different molecular pathways and may be two separate entities [53].

Although our results suggest that the highest level of CDC25B expression seems be important for GC initiation in our population, previous studies described its increased expression associated with advanced tumor stage, deeper invasion, and metastasis in East Asian population [22–24]. As described above, the expression of CDC25B was up-regulated in most of the studied GC samples, despite the invasion or metastasis. Further studies are still required to improve the knowledge about CDC25B function during GC progression; however, it is important to highlight that this is the largest cohort of GC and paired non-neoplastic gastric specimens in which CDC25B expression was evaluated.

Interestingly, even although not statistically significant, YWHAE expression was increased and CDC25B expression was reduced continuously between stages I to and III. However, in the stage IV, YWHAE expression returns to decrease and CDC25B expression returns to increase. In this stage, the highest MYC expression was detected. Here, we observed that only in GC samples with highest *MYC* expression, *MYC* expression was directly correlated with *CDC25B* expression and inversely correlated with *YWHAE* expression, such as observed in GC cell lines.

In GC samples from a Chinese population, a correlation between MYC and CDC25B immunoreactivity was described [25]; however, the effect of tumor stage in the immunoreactivity of these proteins was not accessed. MYC is able to regulate the transcription and hyperactivate cyclin/CDK complexes by the activation of CDC25 phosphatases and CDK kinases [54]. It has been proposed that the synthetic lethality based on the inhibition of CDK may be an interesting method for the treatment of tumors with MYC up-regulation [54]. However, we hypothesized that this approach is promising only in a subset of GC with highest *MYC* expression.

MYC is able to regulate several processes, including apoptosis, proliferation, cell growth, differentiation, angiogenesis, and cell metabolism [55]. Here, *MYC* silencing is able to reduce cancer cell proliferation, invasion and migraion *in vitro* in agreement with previous studies [56–59]. Although we observed that *MYC* induces cell proliferation, GC cells without *MYC* silencing presented more cells at G1 phase than *MYC*-silenced cells after 72 h of culture. This finding is probably due to the rapid accumulation in G1 phase just before confluence observed in controls cells, which therefore were not able to progress through the cell cycle even with active *MYC*.

All studied tumors presented increased *MYC* mRNA expression, and most of the tumors presented increased protein expression in relation to paired non-neoplastic specimens. As described by our group in a smaller cohort [34], MYC increased expression was associated with intestinal-type, deeper tumor extension and the presence of metastasis. Elevated expression of MYC was also associated with late-onset and advanced stage. In a previous study of our group, we reported that *MYC* amplification, which was associated with its immunoreactivity, was associated with these clinicopathological characteristics [34]. Moreover, MYC expression continuously increased during GC progression, with the highest expression detect in stage IV tumors. Thus, MYC deregulated expression is a common finding in GC, especially in intestinal-type and late-onset tumors, and has a role in poor prognosis.

In conclusion, decreasing YWHAE and increasing CDC25B expression seems to be important for tumor development, especially in diffuse-type tumors of early-onset. Conversely, increased MYC expression is a common finding in GC, especially in intestinal-type and late-onset tumors, and has a role in poor prognosis. In GC cell lines, *YWHAE* is able to regulate the GC cell proliferation, invasion and migration through the reduction of *MYC* and *CDC25B* expression. On the other hand, *MYC* also regulates the GC cell proliferation, invasion and migration through the induction of *CDC25B* and the reduction of *YWHAE*. In the tumor initiation, the opposite role of the possible tumor suppressor *YWHAE* and oncogene *CDC25B* in gastric carcinogenesis seems to be independent of *MYC* expression. However, the inversely correlation between *YWHAE* and *MYC* expression seems to be important for GC cells invasion and migration. The inverse correlation between these genes was only detected in a subset of GC, including GC samples at stage IV. Thus, the interaction between YWHAE and MYC and the activation of the pathways related to this interaction may be restricted to a subset of GC and may play a role in the metastasis process.

## MATERIALS AND METHODS

### Cell lines and culture

Three GC cell lines previously established and characterized by our group were used: AGP01, ACP02, and ACP03 [43]. The three cell lines present chromosome 8 trisomy and *MYC* amplification [43, 46].

A cell culture of non-neoplastic gastric mucosa cells (Normal Gastric Mucosa Cell Line 01, MNP01) pooled from 10 patients without gastric cancer was also used to initially evaluate the gene and protein expression of YWHAE, CDC25B, and MYC.

Cell were cultured in Dulbecco's modified Eagle's medium (DMEM; Gibco/Invitrogen, Germany) supplemented with 10% fetal bovine serum (Gibco/Invitrogen, Germany), 100 U/ml penicillin, 100 μg/ml streptomycin, and 0.25 μg/ml amphotericin B. All cultures were maintained in a 5% CO_2_ air-humidified atmosphere at 37°C.

### Gene expression depletion by small interfering RNA (siRNA) transfection

An amount of 3×10^5^ cells were seeded into 6 cm^2^ plates for each cell line before transfection. Cells were cultured for 24 h until cell density was approximately 50%. For *YWHAE* and *CDC25B* silencing, the cells were transfected into AGP01, ACP02, and ACP03 cell lines using either Silencer Select siRNA specific for *YWHAE* (s16; #4390824; Ambion, USA) and *CDC25B* (s2753; #4390824; Ambion, USA), respectively, or the Silencer select negative control #1 (#4390843; Ambion, USA). For *MYC* silencing, a pool of four different double-stranded siRNAs targeting *MYC* (20 μM; SMARTpool ON-TARGETplus MYC siRNA, L-003282-02-0020; GE Healthcare Dharmacon, USA) or scrambled control siRNAs (ON-TARGETplus Nontargeting Pool, D-001810-10-05; GE Healthcare Dharmacon, USA) were transfected into AGP01, ACP02, and ACP03 cell lines using Lipofectamine RNAiMAX Transfection Reagent (Life Technologies, USA).

All siRNA experiments were performed three times.

### Cell proliferation by direct counting

After 24, 48, and 72 h of siRNA transfection, AGP01, ACP02, and ACP03 cells were harvested and directly counted in Neubauer chambers. The total number of cells was estimated and used to determine cell proliferation. Each sample was repeated three times and all experiments were carried in triplicates.

### Cell cycle analysis by flow cytometry

For this analysis, siRNA transfection as carried for 72 h. Cells were treated with 10 μM BrdU for 60 min and then trypsinized and fixed in 80% ethanol at -20 °C overnight. The cell pellet was then treated with 2 M HCl/0.5% Triton X-100 for 30 min at room temperature, neutralized with 0.1 M Na_2_B_4_O_7_, and stained with FITC-anti BrdU antibodies. After centrifugation at 1000 rpm for 5 to 7 min, 400 μL propidium iodide (PI)-RNase solution (final concentrations: 38 mM Na_3_C6H_5_O_7_+69 μM PI+1 μL of 10 mg/mL RNase A) was added to the pellet and mixed well. Samples were incubated at room temperature in the dark for 30 min at 37 °C before analysis by BD FACSCanto™ II (BD Biosciences, USA) flow cytometer. Each sample was repeated three times. The forward light scatter (FSC) of nonfixed cells was used as a relative measure of cell size.

### Invasion and migration analysis

For these analyses, siRNA transfection as carried for 24 h. Invasion assay was done in a 24-well transwell chamber. Cells were added to coated filters in 100 μL of serum-free medium. In the lower compartments of the chambers, 600 μL of human fibroblast serum-free-conditioned media was used as chemo attractant. After 18 h at 37 °C in a 5% CO2 incubator, the Matrigel coating on the upper surface of the filter was wiped off using a cotton swab. Cells that migrated through the filters were fixed, stained with crystal violet, photographed, and counted.

For the migration analysis, cells were loaded on transwell polycarbonate membrane inserts. The plates were incubated for 18 h at 37 °C in a 5% CO2 incubator, the cells in the lower wells were fixed, stained with crystal violet, and counted. The cells that had migrated to the lower compartment of the chambers were trypsinized and counted.

Each experiment was carried out in triplicate.

### Patients and tissue specimens

We enrolled 129 patients with GC who underwent surgical resection with curative intent from Northern Brazil. All patients had negative histories of exposure to either chemotherapy or radiotherapy before surgery. Patients with co-occurrence of other diagnosed cancers were excluded from this study. Signed informed consent was obtained from all patients before sample collection. The study protocol was approved by the Ethics Committee of the Hospital Universitário João de Barros Barreto (Protocol #316737).

Part of each dissected tumor sample was formalin fixed and paraffin embedded (FFPE). Sections of the FFPE tissue were stained with hematoxylin and eosin for histological evaluation or used for immunohistochemical (IHC) analysis. Additional portions of each tumor and paired non-neoplastic tissue specimens were snap frozen in liquid nitrogen and stored at -80 °C until protein and nucleic acid purification.

All samples were classified according to Laurén [60] and the tumors were staged according to the tumor-node-metastasis (TNM) staging criteria [61]. The presence of *Helicobacter pylori* in gastric samples was detected by the rapid urease test, and its virulence factor cytotoxicity-associated gene A (CagA gene) was detect by polymerase chain reaction (PCR) using DNA purified simultaneously with proteins and mRNA, as previously performed by our group [62]. Epstein-Barr virus (EBV) was detected by RNA *in situ* hybridization [62].

### DNA/RNA/protein purification

Total RNA and proteins were extracted with TRIzol reagent from GC cell lines after 48 h of transfection. Total protein, mRNA, and DNA were simultaneously isolated from gastric tissue samples using the AllPrep DNA/RNA/Protein Kit (Qiagen, Germany) according to the manufacturer's instructions. The protein pellet was dissolved in a buffer containing 7 M urea, 2 M thiourea, 4% CHAPS, 50 mM DTT, 1% Protease Inhibitor Cocktail (Sigma-Aldrich, USA), and 0.5% each of Phosphatase Inhibitor Cocktail 1 and 2 (Sigma-Aldrich, USA), as previously performed by our group [15]. The protein concentrations were determined by the method of Bradford (Sigma-Aldrich, USA). The RNA concentration and quality were determined using a NanoDrop spectrophotometer (Kisker, Germany) and 1% agarose gels, respectively. Samples were stored at -80 °C until use.

### mRNA expression

RNA was reverse transcribed using the Reverse Transcription System according to the manufacturer's protocol (A3500; Promega, USA). Complementary DNA was then amplified by real-time reverse transcription quantitative PCR (RT-qPCR) using TaqMan probes purchased as Assays-on-Demand Products for Gene Expression (Life Technologies, USA) and a 7500 Fast Real-Time PCR instrument (Life Technologies, USA). The *ACTB* gene was selected as an internal control [63]. All RT-qPCRs were performed in triplicate for both the target genes (*YWHAE:* Hs00356749_g1; *CDC25B*: Hs00244740_m1; *MYC*: Hs00153408_m1) and the internal control (*ACTB*: 4333762F).

The relative quantification (RQ) of gene expression was calculated according to Livak and Schmittgen [64]. In tissue sample analyses, the corresponding control sample was designated as a calibrator from each tumor. In the cell line analysis, the siRNA control-transfected cells were used as a calibrator. The gene expression in the MNP01 was also designated as a calibrator from all GC cell lines.

### Western blotting

Western blot analysis was performed as described previously [15]. Reduced protein (25 μg) from each sample was separated by 12.5% homogeneous sodium dodecyl sulfate-polyacrylamide gel electrophoresis (SDS-PAGE) and electroblotted onto a polyvinylidene fluoride (PVDF) membrane (Hybond-P; GE Healthcare, USA). The PVDF membrane was blocked with phosphate-buffered saline containing 0.1% Tween 20 and 5% low fat milk and incubated overnight at 4 °C with the corresponding primary antibodies: anti-YWHAE (dilution 1:1000; PA5-29773; Life Technologies, USA), anti-CDC25B (dilution 1:1000; PA5-14100; Life Technologies, USA), anti-MYC (dilution 1:50; MA5-12080; Life Technologies, USA), and anti-ACTB (dilution 1:250; Ac-15; Life Technologies, USA). After extensive washing, a peroxidase-conjugated secondary antibody was added for 1 h at room temperature. Immunoreactive bands were visualized using the Western blotting Luminol reagent, and the images were acquired using an ImageQuant 350 digital image system (GE Healthcare, Sweden). ACTB was used as a loading reference control.

### Immunohistochemical staining and scoring

Tumor tissue sections (3 or 4 mm thick) were deparaffinized in xylene and rehydrated in a graded series of ethanol. After heat-induced epitope retrieval, the tissue sections were incubated with primary mouse monoclonal antibodies against YWHAE (dilution 1:100; PA5-29773; Life Technologies, USA), CDC25B (dilution 1:10; PA5-14100; Life Technologies, USA) or MYC (dilution 1:100; MA5-12080; Life Technologies, USA). A universal peroxidase-conjugated secondary antibody kit (LSAB System; DakoCytomation, USA) was used for detection. We used 3,3’-diaminobenzidine/H_2_O_2_ (DakoCytomation, Denmark) as the chromogen and hematoxylin as the counterstain. A protein immunoreactivity-positive sample was defined as one having 10% or more neoplastic cells that were positive for the protein.

### Statistical analysis

The data are shown as the frequency, median, and IQR. The Shapiro-Wilk test was used to evaluate the distribution of the age, mRNA, and protein expression data and to determine the appropriate subsequent test for statistical comparisons. The Mann-Whitney test was used to investigate the possible associations between gene mRNA or protein expression and categorical variables, such as immunoreactivity and clinicopathological features. An association between categorical variables was analyzed using the χ^2^ test. The K-means clustering method was used to group samples based on their gene expression similarities. A Spearman correlation test was used to evaluate the possible correlation between mRNA and protein expression. *p*≤0.05 was considered significant. The Bonferroni adjustment of the *p* value was applied when multiple comparisons were performed, with the α level being divided by the number of comparisons.

## SUPPLEMENTARY MATERIALS FIGURES AND TABLES




