# Supplementary material for: YWHAE silencing induces cell proliferation, invasion and migration through the up-regulation of CDC25B and MYC in gastric cancer cells: new insights about YWHAE role in the tumor development and metastasis process
Source: Oncotarget. 2016 Nov 16;7(51):85393–410. doi: 10.18632/oncotarget.13381 (PMC5356744; doi:10.18632/oncotarget.13381)
Supplement: Supplementary file 1 [file oncotarget-07-85393-s001.pdf]

YWHAE silencing induces cell proliferation, invasion and migration through the up-regulation of CDC25B and MYC in gastric cancer cells: new insights about YWHAE role in the tumor development and metastasis process

SUPPLEMENTARY TABLE

Supplementary Table S1: Clinicopathological variables and gene expression clusters in GC

Variable	N	Cluster		*p* value[a]
		#1 [N (%)]	#2 [N (%)]	
**Gender**				
Female	45	22 (48.9)	23 (51.1)	0.326
Male	84	46 (54.8)	38 (45.2)	
**Onset**				
< 45 years	34	22 (64.7)	12 (35.3)	0.076
≥ 45 years	95	46 (48.4)	49 (51.6)	
**Tumor location**				
Cardia	50	29 (58)	21 (42)	0.219
Non-cardia	79	39 (49.4)	40 (50.6)	
**Histological type**				
Diffuse	62	33 (53.2)	29 (46.8)	0.526
Intestinal	67	35 (52.2)	32 (47.8)	
**Stage**				
Early	12	10 (83.3)	2 (16.7)	0.024*
Advanced	117	58 (49.6)	59 (50.4)	
**Tumor invasion**				
T1/T2	42	34 (81.0)	8 (19)	<0.001*
T3/T4	87	34 (39.1)	53 (60.9)	
**Lymph node metastasis**				
Absent	16	16 (100)	0 (0)	<0.001*
Present	113	52 (46)	61 (54)	
**Distant metastasis**				
Absent	70	61 (87.1)	9 (12.9)	<0.001*
Present	59	7 (11.9)	52 (88.1)	
H. pylori				
Negative	13	8 (61.5)	5 (38.5)	0.354
Positive	116	60 (51.7)	56 (48.3)	
**CagA**				
Negative	46	24 (52.2)	22 (47.8)	0.537
Positive	83	44 (53.0)	39 (47.0)	
**EBV**				
Negative	108	60 (55.6)	48 (44.4)	0.110
Positive	21	8 (38.1)	13 (61.9)	

[a]*p* value by χ^2 test. **p*<0.05, significantly difference between groups. N: number of samples; EBV: *Epstein-Barr virus.*
