# Supplementary material for: YWHAE silencing induces cell proliferation, invasion and migration through the up-regulation of CDC25B and MYC in gastric cancer cells: new insights about YWHAE role in the tumor development and metastasis process
Source: Oncotarget. 2016 Nov 16;7(51):85393–410. doi: 10.18632/oncotarget.13381 (PMC5356744; doi:10.18632/oncotarget.13381)
Supplement: Supplementary file 2 [file oncotarget-07-85393-s002.docx]

**Table 1.** Clinicopathological variables and gene expression in GC.

| **Variable** | **N** | **YWHAE immunoreactivity** | | **YWHAE protein** | | ***YWHAE* mRNA** | | **CDC25B immunoreactivity** | | **CDC25B protein** | | ***CDC25B* mRNA** | | **MYC immunoreactivity** | | **MYC protein** | | ***MYC* mRNA** | |
| --- | --- | --- | --- | --- | --- | --- | --- | --- | --- | --- | --- | --- | --- | --- | --- | --- | --- | --- | --- |
|  |  | N (%) of positive cases | *p* value^a^ | Ratio T/N [median (IQR)] | *p* value^b^ | RQ [median (IQR)] | *p* value^b^ | N (%) of positive cases | *p* value^a^ | Ratio T/N [median (IQR)] | *p* value^b^ | RQ [median (IQR)] | *p* value^b^ | N (%) of positive cases | *p* value^a^ | Ratio T/N [median (IQR)] | *p* value^b^ | RQ [median (IQR)] | *p* value^b^ |
| **Gender** |  |  |  |  |  |  |  |  |  |  |  |  |  |  |  |  |  |  |  |
| Female | 45 | 2 (4.4) | 0.652 | 0.57 (0.26) | 0.046* | 0.64 (0.32) | 0.592 | 45 (100) | 0.651 | 1.71 (0.73) | 0.349 | 1.68 (0.61) | 0.376 | 41 (91.1) | 0.589 | 2.11 (1.55) | 0.687 | 3.22 (1.38) | 0.953 |
| Male | 84 | 4 (4.8) |  | 0.49 (0.33) |  | 0.69 (0.30) |  | 83 (98.8) |  | 1.61 (0.66) |  | 1.61 (0.60) |  | 76 (90.5) |  | 2.16 (1.18) |  | 3.11(1.09) |  |
| **Onset** |  |  |  |  |  |  |  |  |  |  |  |  |  |  |  |  |  |  |  |
| < 45 years | 34 | 3 (8.8) | 0.187 | 0.41 (0.27) | 0.006* | 0.49 (0.21) | <0.001* | 34 (100) | 0.736 | 1.96 (0.50) | 0.003* | 1.88 (0.40) | 0.005* | 2 (94.1) | 0.339 | 1.80 (0.92) | 0.013* | 3.04 (1.27) | 0.048* |
| ≥ 45 years | 95 | 3 (3.2) |  | 0.57 (0.29) |  | 0.71 (0.23) |  | 94 (99.2) |  | 1.45 (0.61) |  | 1.57 (0.52) |  | 85 (89.5) |  | 2.23 (1.27) |  | 3.28 (1.01) |  |
| **Tumor location** |  |  |  |  |  |  |  |  |  |  |  |  |  |  |  |  |  |  |  |
| Cardia | 50 | 1 (2) | 0.247 | 0.57 (0.25) | 0.379 | 0.68 (0.22) | 0.489 | 50 (100) | 0.612 | 1.64 (0.64) | 0.625 | 1.61 (0.53) | 0.383 | 43 (86) | 0.126 | 1.87 (1.27) | 0.088 | 3.06 (1.32) | 0.197 |
| Non-cardia | 79 | 5 (6.3) |  | 0.49 (0.34) |  | 0.66 (0.35) |  | 78 (98.7) |  | 1.62 (0.72) |  | 1.68 (0.64) |  | 74 (93.7) |  | 2.20 (1.16) |  | 3.23 (0.93) |  |
| **Histological type** |  |  |  |  |  |  |  |  |  |  |  |  |  |  |  |  |  |  |  |
| Diffuse | 62 | 3 (4.8) | 0.622 | 0.39 (0.25) | <0.001* | 0.58 (0.33) | 0.019* | 62 (100) | 0.519 | 1.84 (0.72) | 0.041* | 1.78 (0.62) | 0.027* | 52 (83.9) | 0.010* | 2.20 (1.13) | 0.786 | 3.21 (0.92) | 0.854 |
| Intestinal | 67 | 3 (4.5) |  | 0.63 (0.22) |  | 0.72 (0.26) |  | 66 (98.5) |  | 1.43 (0.57) |  | 1.52 (0.50) |  | 65 (97) |  | 2.11 (1.40) |  | 3.09 (1.22) |  |
| **Stage** |  |  |  |  |  |  |  |  |  |  |  |  |  |  |  |  |  |  |  |
| Early | 12 | 0 (0) | 0.550 | 0.36 (0.28) | 0.027* | 0.48 (0.19) | 0.003* | 12 (100) | 0.907 | 1.83 (0.45) | 0.102 | 1.80 (0.33) | 0.046* | 10 (83.3) | 0.309 | 1.37 (0.60) | <0.001* | 2.25 (1.25) | 0.003* |
| Advanced | 117 | 6 (5.1) |  | 0.55 (0.29) |  | 0.68 (0.29) |  | 116 (99.1) |  | 1.61 (0.68) |  | 1.62 (0.60) |  | 107 (91.5) |  | 2.19 (1.24) |  | 3.23 (1.03) |  |
| **Tumor invasion** |  |  |  |  |  |  |  |  |  |  |  |  |  |  |  |  |  |  |  |
| T1/T2 | 42 | 0 (0) | 0.089 | 0.42 (0.28) | 0.006* | 0.57 (0.23) | 0.001* | 42 (100) | 0.674 | 1.79 (0.42) | 0.004* | 1.76 (0.33) | 0.004* | 36 (85.7) | 0.151 | 1.63 (0.77) | <0.001* | 2.59 (1.12) | <0.001* |
| T3/T4 | 87 | 6 (6.9) |  | 0.58 (0.28) |  | 0.72 (0.29) |  | 86 (98.9) |  | 1.35 (0.67) |  | 1.48 (0.62) |  | 81 (93.1) |  | 2.38 (1.20) |  | 3.40 (0.95) |  |
| **Lymph node metastasis** |  |  |  |  |  |  |  |  |  |  |  |  |  |  |  |  |  |  |  |
| Absent | 16 | 1 (6.3) | 0.556 | 0.52 (0.27) | 0.379 | 0.62 (0.27) | 0.438 | 15 (93.8) | 0.124 | 1.93 (0.65) | 0.092 | 1.86 (0.58) | 0.123 | 11 (68.8) | 0.007* | 1.42 (0.25) | <0.001* | 2.36 (0.48) | <0.001* |
| Present | 113 | 5 (4.4) |  | 0.55 (0.31) |  | 0.67 (0.32) |  | 113 (100) |  | 1.61 (0.65) |  | 1.62 (0.59) |  | 106 (93.8) |  | 2.23 (1.21) |  | 3.29 (0.97) |  |
| **Distant metastasis** |  |  |  |  |  |  |  |  |  |  |  |  |  |  |  |  |  |  |  |
| Absent | 70 | 3 (4.3) | 0.576 | 0.57 (0.30) | 0.134 | 0.67 (0.27) | 0.970 | 69 (98.6) | 0.543 | 1.63 (0.69) | 0.620 | 1.63 (0.59) | 0.712 | 58 (82.9) | <0.001* | 1.67 (0.61) | <0.001* | 2.63 (0.76) | <0.001* |
| Present | 59 | 3 (5.1) |  | 0.49 (0.32) |  | 0.66 (0.36) |  | 59 (100) |  | 1.63 (0.68) |  | 1.67 (0.64) |  | 59 (100) |  | 2.93 (0.96) |  | 3.74 (0.44) |  |
| ***H. pylori*** |  |  |  |  |  |  |  |  |  |  |  |  |  |  |  |  |  |  |  |
| Negative | 13 | 0 (0) | 0.522 | 0.39 (0.31) | 0.109 | 0.57 (0.35) | 0.315 | 13 (100) | 0.899 | 1.76 (0.82) | 0.134 | 1.72 (0.68) | 0.127 | 12 (92.3) | 0.654 | 2.17 (1.41) | 0.879 | 3.02 (1.27) | 0.656 |
| Positive | 116 | 6 (5.2) |  | 0.55 (0.31) |  | 0.67 (0.30) |  | 115 (99.1) |  | 1.62 (0.68) |  | 1.63 (0.60) |  | 105(90.5) |  | 2.14 (1.30) |  | 3.21 (1.16) |  |
| **CagA** |  |  |  |  |  |  |  |  |  |  |  |  |  |  |  |  |  |  |  |
| Negative | 46 | 0 (0) | 0.066 | 0.54 (0.32) | 0.611 | 0.66 (0.30) | 0.508 | 45 (97.8) | 0.357 | 1.63 (0.71) | 0.526 | 1.63 (0.55) | 0.665 | 41 (89.1) | 0.435 | 2.08 (1.15) | 0.789 | 3.17 (1.05) | 0.904 |
| Positive | 83 | 6 (7.2) |  | 0.56 (0.31) |  | 0.70 (0.33) |  | 83 (100) |  | 1.64 (0.69) |  | 1.65 (0.66) |  | 76 (91.6) |  | 2.18 (1.33) |  | 3.17 (1.14) |  |
| **EBV** |  |  |  |  |  |  |  |  |  |  |  |  |  |  |  |  |  |  |  |
| Negative | 108 | 4 (3.7) | 0.252 | 0.54 (0.30) | 0.742 | 0.67 (0.30) | 0.723 | 107 (99.1) | 0.837 | 1.64 (0.69) | 0.730 | 1.64 (0.57) | 0.990 | 96 (88.9) | 0.107 | 2.04 (1.14) | 0.024* | 3.08 (1.10) | 0.025* |
| Positive | 21 | 2 (9.5) |  | 0.58 (0.36) |  | 0.64 (0.34) |  | 21 (100) |  | 1.39 (0.68) |  | 1.60 (0.66) |  | 21 (100) |  | 2.57 (1.71) |  | 3.69 (1.19) |  |

^a^*p* value by χ^2^ test; ^b^*p* value by Mann-Whitney test. **p*<0.05, significantly difference between groups. **A tendency for different expression between groups. N: number of samples; T/N: ratio of protein expression between neoplastic and matched non-neoplastic samples; RQ: relative quantification, in which the matched non-neoplastic sample was designated as a calibrator from each neoplastic samples; IQR: interquartile range; EBV: *Epstein-Barr virus.*
